# Enhancing DNA recovery in low-biomass snow algae samples: a comparative study of extraction methods and their effect on community composition

**DOI:** 10.1128/aem.00031-26

**Published:** 2026-03-19

**Authors:** P. Almela, T. L. Hamilton

**Affiliations:** 1 Department of Plant and Microbial Biology, University of Minnesota 172728 , St. Paul, Minnesota, USA; Universidad de los Andes, Bogotá, Colombia

**Keywords:** amplicon sequencing, extraction method, snow algae, bacterial community, eukaryal community

## Abstract

High-throughput sequencing is a powerful tool for environmental microbiology and can be particularly important for examining community structure and function for organisms that are difficult to culture or environments that are difficult to mimic, like snow. Nucleic acid extraction significantly impacts these analyses, often introducing more variation between samples than PCR or sequencing. Snow algae are widespread on mountain and polar snowfields, where they contribute to biogeochemical cycling and accelerate melt. Despite increasing research on snow algae, DNA extraction remains challenging, as the thick, resilient walls of snow algal cysts can limit cell lysis, and differences among extraction methods may therefore affect the estimates of community composition and richness. Here, we compared three common extraction methods (Qiagen DNeasy PowerSoil Pro, Qiagen DNeasy PowerWater, and phenol-chloroform) alongside ultrasonication in samples with varying snow algae abundance. The extraction method strongly influenced the resulting microbial profiles assessed by amplicon sequencing of rRNA genes. Ultrasonication improved DNA yield in low-biomass samples and enhanced the recovery of DNA from resilient cells, including mature-phase snow algae, likely due to improved cell lysis. Our findings provide insights to improve standardization and facilitate comparison among studies in snow and ice environments.

**IMPORTANCE:**

High-throughput sequencing has transformed environmental microbiology, allowing for detailed, culture-independent analyses of microbial communities. However, multiple methodological factors, including DNA extraction, can introduce variability in results, making cross-study comparisons challenging. This research contributes to improving our understanding of snow algae, which play a role in alpine and polar ecosystems by influencing biogeochemical cycles and snow reflectivity. By evaluating common DNA extraction techniques for snow algae, this study helps improve the reliability and reproducibility of sequencing data, supporting broader efforts toward methodological standardization in microbial ecology.

## INTRODUCTION

High-throughput sequencing has revolutionized environmental microbiology by enabling rapid, culture-independent analyses of microbial communities, providing insights into their composition, function, and dynamics across diverse environments (1–4). However, sequencing-based community analyses are sensitive to methodological choices at multiple stages of the workflow, each introducing potential sources of bias. These include DNA extraction and isolation, primer selection and PCR conditions, sequencing platforms, sequence clustering approaches (amplicon sequence variants [ASVs] vs. operational taxonomic units [OTUs]), and reference database accuracy (5–10). Among these steps, the choice of DNA extraction method has been shown to be a major source of variation in inferred community composition (11–16), in part because low DNA yields can exacerbate PCR-related biases and alter the observed community profiles (17). The absence of standardized methodologies throughout the entire process may further contribute to variability in results across studies, affecting the comparability and reproducibility of findings.

Snow algae are photosynthetic microorganisms (Chlorophyta) that thrive in extreme cold environments, forming characteristic red, green, or orange blooms on snow surfaces (18). These blooms play an important role in alpine and polar ecosystems, influencing biogeochemical cycles and altering snow reflectivity (19–21). Although snow algae communities are increasingly studied using amplicon sequencing, there is no standardized methodology for their analysis, making comparisons between studies challenging. Studies often employ different DNA extraction methods, including both commercial (e.g., 22–26) and non-commercial methods (e.g., 27, 28), which may introduce variability in results.

Although snow is fundamentally solid water, snowpacks with algal blooms represent a distinct environmental matrix that may be enriched with a combination of organic and inorganic materials that can interfere with DNA extraction. Because commercial extraction kits are typically optimized for either aqueous samples (e.g., biomass collected on a filter) or soil samples, selecting an effective strategy for snow algae remains non-trivial. Accordingly, differences in DNA extraction strategies in recent snow algae studies (22, 23, 27, 28) may impact the comparison of results, as previously observed in bacterial community analysis from the human microbiome (29). Yan et al. (13) evaluated DNA extraction methods for assessing bacterial diversity in snow, but differences in cellular structure between bacteria and snow algae limit the transferability of those findings, and no study to date has systematically assessed the performance of commonly used DNA extraction methods for snow algae. Identifying the most suitable extraction method for snow algae is crucial for generating reliable and reproducible DNA sequencing data and for advancing efforts toward methodological standardization.

Here, we evaluated the efficiency of two commercially available Qiagen DNA extraction kits: the DNeasy PowerSoil Pro Kit, a commonly used approach in snow-algae studies (30–34), and the DNeasy PowerWater Kit, which is more commonly applied in aquatic microbiome studies (35–37). These kits were selected to explicitly test whether soil- or water-optimized extraction strategies are better suited for snow algae samples. For comparison, we also included a phenol-chloroform extraction, a classical and broadly applied method in molecular biology (e.g., 38, 39), which serves as a chemistry-based benchmark independent of commercial kit design.

We assessed DNA yield, community composition, and richness to gain a more detailed understanding of method-specific biases. Analyses were conducted on the total microbial community, as well as separately on bacterial, eukaryotic, and specifically algal assemblages. By applying these extraction protocols to field samples spanning contrasting snow algae cell abundance, this study aims to identify methodological biases that influence amplicon sequencing outcomes and to provide guidance toward best practices for short-read high-throughput sequencing studies of snow algae diversity.

## MATERIALS AND METHODS

### Sample collection and biomass estimations

Two samples with different cell densities per surface area (i.e., biomass), initially determined visually in the field based on color intensity, were used in this study. A “high-biomass” sample was collected on June 25, 2024, from a seasonal snowfield at Brian Head, Utah (37°41'08.4"N, 112°49'25.5"W), while a “low-biomass” sample was collected on July 2, 2024, from a seasonal snowfield at Mount Shasta, California (41°22'05.5"N, 122°11'53.9"W).

At each site, a 36 × 36 × 7 cm snow plot was collected, placed in a sterile plastic bag, and transported to the laboratory within 2–3 h. After melting, 45 mL subsamples were transferred into sterile 50-mL tubes, wrapped in aluminum foil, and stored at 4°C for 1 month prior to analysis. Before further processing, two 45 mL subsamples were combined and homogenized. Twenty-five 2 mL aliquots per plot were filtered onto ashed 0.7-µm pore size Whatman GF/F filters, wrapped in aluminum foil, and frozen at −20°C until DNA extractions. To estimate biomass from cell counts, a 10 mL aliquot of melted snow was collected in a sterile 15-mL conical tube and amended 4% Lugol’s solution. Cell counts were performed using 9–16 replicate measurements in a hemocytometer chamber (Hausser Scientific) under a light microscope (Leitz LaborLux S, 10× objective).

### DNA extraction methods

Seven DNA extraction methods were tested on the low (500 cells·mL⁻¹) and high (~16·10^4^ cells·mL⁻¹) snow algae biomass samples, using three replicates per method. Methods 1–3 (DNeasy PowerSoil Pro Kit, Qiagen) and Methods 4 and 5 (PowerWater Kit, Qiagen) were based on commercial protocols but with modifications in the homogenization and cell lysis steps. Methods 6 and 7 were non-commercial approaches based on the traditional phenol-chloroform extraction technique but with altered homogenization and cell lysis steps. The key characteristics of the different methods are summarized in Table 1 and briefly below.

**TABLE 1:** Overview of the main features of the seven selected DNA extraction methods^*a*^

	Method 1	Method 2	Method 3	Method 4	Method 5	Method 6	Method 7
Sample prep	PowerBead Pro tubes	PowerBead Pro tubes	PowerBead Pro tubes	Silica Spin Filter Tubes	Silica Spin Filter Tubes	–^*b*^	–
Homogenization	Vortex (10 min)	Homogenizer (2 × 30 s)	Sonication (50A, 1 min)	Homogenizer (2 × 30 s)	Sonication (50A, 1 min)	–	Sonication (50A, 1 min)
Cell lysis	Solution CD1	Solution CD1	Solution CD1	Solution PW1	Solution PW1	Lysis Buffer Tris-HCl, EDTA, SDS pH 8.0 Mild vortex Incubation at 37°C Proteinase K addition and incubation at 70°C	Lysis Buffer Tris-HCl, EDTA, SDS pH 8.0 Mild vortex Incubation at 37°C Proteinase K addition and incubation at 70°C
Purification	Inhibitor Removal Technology (CD2)	Inhibitor Removal Technology (CD2)	Inhibitor Removal Technology (CD2)	Inhibitor Removal Technology (IRS)	Inhibitor Removal Technology (IRS)	Phenol:Chloroform:Isoamyl alcohol (×1) Chloroform:Isoamyl alcohol (×1)	Phenol:Chloroform:Isoamyl alcohol (×1) Chloroform:Isoamyl alcohol (×1)
Precipitation	Column binding and cleaning (CD3, EA, C5)	Column binding and cleaning (CD3, EA, C5)	Column binding and cleaning (CD3, EA, C5)	Column binding and cleaning (PW3, PW4, PW5)	Column binding and cleaning (PW3, PW4, PW5)	Linear Polyacrylamide Sodium acetate Isopropanol −20°C	Linear Polyacrylamide Sodium acetate Isopropanol −20°C

^*a*^ The table summarizes key attributes and specific characteristics of each method tested.

^*b*^ –, not applicable.

Methods 1, 2, and 3 followed the manufacturer’s recommended sequence of treatments and washes using the standard buffers from the DNeasy PowerSoil Pro Kit, ultimately eluting the DNA from silica columns with 60 µL of elution buffer. Different homogenization methods were tested for Method 1 and Method 2, following steps 2a and 2b of the DNeasy PowerSoil Pro Kit Handbook. Step 2a specifies vortexing at maximum speed for 10 min, while step 2b involves homogenization for 30 s, followed by a 30-s pause and a second homogenization for another 30 s. In Method 3, ultrasonication (50A, 1 min) was applied as an additional pre-treatment to enhance cell resuspension and fragmentation, as suggested by Tucker and Brown (34); this step did not replace mechanical lysis, and samples subsequently underwent the standard bead-beating/homogenization step of the DNeasy PowerSoil Pro Kit as recommended by the manufacturer, followed by the standard washes and elution.

In Methods 4 and 5, we followed the manufacturer’s recommended protocol from the DNeasy PowerWater Kit, a commercial kit previously used for microalgae (35). In Method 4, an additional lysis step at 65°C for 10 min was performed as part of the extraction as recommended by the supplier for hard-to-lyse samples. In Method 5, ultrasonication was performed (50A, 1 min) following the additional 65°C for 10 min lysis step. Finally, DNA was eluted from the silica columns using 60 µL of elution buffer.

Methods 6 and 7 involved chemical and enzymatic treatment of samples according to Busi et al. (36), with minor modifications from Green and Sambrook (37). For both methods, filters were submerged in 10 mL of lysis buffer (0.1 M Tris-HCl pH 7.5, 0.05 M EDTA pH 8, 1.25% SDS). Methods 6 and 7 followed identical protocols, with the only difference being an additional sonication step in Method 7 (50 A, 1 min). Following lysis (or lysis and ultrasonication), 10 µL of RNase A (100 mg/mL) was added. Samples were vortexed for 15 s and incubated at 37 °C for 1 h with rotation. Subsequently, 100 µL of Proteinase K (20 mg/mL) was added, and samples were incubated statically for 10 min at 70°C. Samples were extracted once with phenol/chloroform/isoamyl alcohol (25:24:1), followed by extraction of the supernatant with chloroform/isoamyl alcohol (24:1). For both methods, more stringent DNA precipitation conditions were applied by adding 10 µg/mL LPA and incubating overnight at −20 °C.

The extracted DNA was stored at −20°C until further analysis. The concentration of DNA was determined using the Qubit dsDNA HS kit (Invitrogen) and a Qubit 3.0 Fluorometer (Life Technologies, Carlsbad, CA, USA). DNA quality was assessed by visualizing samples on a 0.8% agarose gel containing GelRed nucleic acid stain.

### DNA extraction controls

A 1-mL aliquot of a snow algae culture (*Chloromonas typhlos* CCAP11/128) in the exponential stage (146 × 10⁴ cells·mL⁻¹), dominated by actively growing, flagellated vegetative cells, was used as a positive control. This control allowed us to compare the extraction efficiency between field samples containing mature snow algae with resistant cyst stages (38) and lab-grown cells that are likely easier to lyse. Extractions were evaluated based on the concentration of DNA recovered.

Extraction blanks—tubes processed without any sample—were used to check for contamination. Each negative underwent the same extraction procedures as the test samples. All negative controls were below the limit of detection in Qubit assays (HS kit, Invitrogen), and no DNA was visible in negative controls when assessed using agarose gel electrophoresis.

### Amplicon sequencing

Sequencing was performed on low-biomass samples because they represent the most constraining condition for DNA-based analyses, where insufficient DNA yield is most likely to limit library preparation and require additional PCR amplification, potentially introducing amplification biases. Only DNA extracted with the DNeasy PowerSoil Pro Kit or the phenol-chloroform method (Methods 1, 2, 3, 6, and 7) was sequenced because these methods yielded the highest DNA quantity and quality for downstream analyses. The PowerWater kit was excluded from downstream analysis due to lower DNA concentration and quality. Amplicon library preparation and sequencing were performed at the University of Minnesota Genomics Center (UMGC) using an Illumina platform, generating 2 × 250 bp paired-end reads. Eukaryotic DNA was amplified using primers 1391f and EukBr targeting the V9 region of the 18S SSU rRNA, following protocols based on previous studies (39, 40). Prokaryotic DNA was amplified using primers 515F–806R targeting the V4 region of the 16S SSU rRNA (41).

### 16S and 18S rRNA amplicon analysis

Diversity and community composition were assessed with QIIME v2-2024.10 (42). Briefly, cleaned and trimmed paired reads were filtered and denoised using the DADA2 plug-in (43). For chimera identification, 340,000 training sequences were used. Identified operational taxonomic units (OTU), defined at 97% of similarity, were aligned using MAFFT (44) and further processed to construct a phylogeny with fasttree2 (45). Taxonomy was assigned to OTUs using the q2-feature-classifier (46) and BLASTN against the SILVA v138 99% sequence database (47).

### Data analysis and statistics

Figures showing DNA concentration, total sequence counts, and diversity indices were generated using GraphPad Prism (v8.3.0). Differences in microbial community composition at the phylum, family, and OTU levels were evaluated using permutational multivariate analysis of variance (PERMANOVA). Principal coordinates analysis (PCoA) based on Bray-Curtis dissimilarities was performed in R using the vegan package (48) to visualize similarities among extraction methods. In addition, a UPGMA cluster dendrogram based on Bray-Curtis dissimilarity was generated using PAST software (v4.12) to further explore community similarity across methods. In the case of the snow algae community, taxonomic differences were evaluated at the genus level, with manual assignments conducted using BLASTN (49). After performing the BLASTN analyses, OTUs that showed the highest match with the same reference sequence were grouped together to refine the taxonomic classification and community composition. DESeq2 (50) was used to assess significant differences in snow algae community abundances at the genus level between methods. Log₂-fold changes were calculated to contrast phenol-chloroform extractions (Methods 6 and 7) against commercial kits (Methods 1–3) and to evaluate the effect of ultrasonication by comparing sonicated protocols (Methods 3 and 7) with non-sonicated protocols (Methods 1, 2, and 6).

## RESULTS

### DNA concentration in low-biomass samples

**Fig 1:**
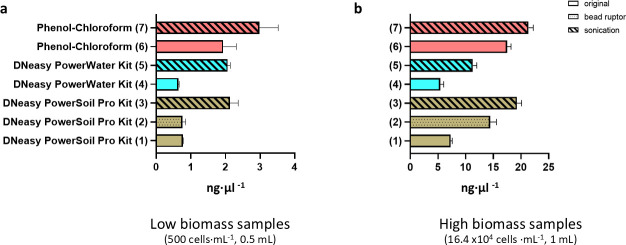
DNA concentrations measured by Qubit for snow algae samples with low biomass (**a**) and high biomass (**b**), comparing different extraction methods.

For low-biomass samples (500 cells·mL⁻¹; 0.5 mL), some DNA extraction methods yielded limited DNA quantities that can constrain downstream library preparation and sequencing, given the minimum input requirements imposed by sequencing facilities. For Methods 1, 2, and 4, DNA yields did not exceed 1 ng·µL⁻¹ and totaled less than 60 ng (Fig. 1a), and no significant differences were observed among these methods, which differed only in the commercial kit used (DNeasy PowerSoil Pro vs. DNeasy PowerWater) and/or the homogenization approach.

Incorporating ultrasonication during DNA extraction significantly enhanced DNA yield compared to standard methods (Table S1). For instance, Method 5, DNeasy PowerWater Kit and ultrasonication (2.1 ± 0.1 ng·µL⁻¹), resulted in a 224.6% increase in yield compared to Method 4 (0.6 ± 0.0 ng·µL⁻¹). Similarly, using the DNeasy PowerSoil Pro Kit and ultrasonication, DNA yields in Method 3 (2.1 ± 0.2 ng·µL⁻¹) improved by 175.9% and 183.2% relative to Method 1 (0.8 ± 0.0 ng·µL⁻¹) and 2 (0.8 ± 0.1 ng·µL⁻¹), respectively.

Method 6 (1.9 ng·µL⁻¹) generated over twice the DNA yield compared to the two commercial methods following standard protocols. Ultrasonication further enhanced DNA recovery (Fig. 1a), with Method 7 yielding the highest concentration (3.0 ± 0.5 ng·µL⁻¹), representing a 58.0% increase over Method 6. Although commercial kits combined with ultrasonication (Methods 3 and 5) produced higher yields than the average in the standard phenol-chloroform method, they remained significantly lower than those obtained with Method 7.

### DNA concentration in high-biomass samples

When analyzing samples with a high-biomass concentration (16.4 × 10⁴ cells·mL⁻¹; 1 mL), significant differences in DNA yield were observed across extraction methods. For Methods 1 and 2 (Fig. 1b), the choice of homogenization technique had a considerable impact, with DNA yield doubling when homogenization was used instead of vortexing (7.3 ± 0.3 ng·µL⁻¹ vs. 14.4 ± 1.1 ng·µL⁻¹, respectively). The incorporation of ultrasonication further improved DNA extraction efficiency across all methods. Method 3 (19.3 ± 0.9 ng·µL⁻¹) resulted in a 164% increase in DNA yield compared to Method 1 and a 33.5% increase compared to Method 2. Similarly, for the DNeasy PowerWater Kit, DNA yield in Method 5 (11.3 ± 0.7 ng·µL⁻¹) was 109.0% higher than in Method 4 (5.4 ± 0.6 ng·µL⁻¹).

Method 6 yielded the highest DNA concentration (17.5 ± 0.7 ng·µL⁻¹) among all methods, producing more than twice the amount obtained with Method 4 (5.4 ng·µL⁻¹) using commercial kits. Incorporating ultrasonication into the phenol-chloroform extraction led to a ~20% improvement in DNA recovery (21.4 ± 0.9 ng·µL⁻¹ for Method 7), demonstrating its effectiveness in enhancing extraction efficiency across different methods.

### DNA quality in low- and high-biomass samples

**Fig 2:**
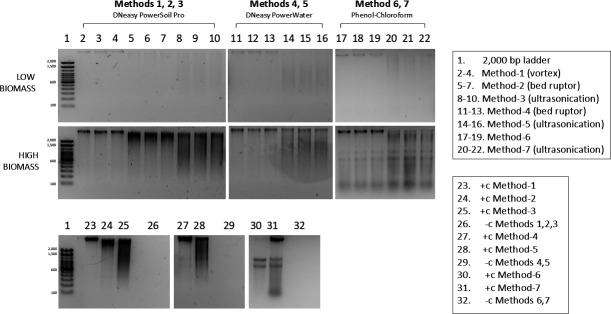
Agarose gel electrophoresis (1% agarose) of genomic DNA from low- and high-biomass snow algae samples extracted using different methods. Methods 1–3 used the DNeasy PowerSoil Pro Kit (Qiagen), Methods 4 and 5 used the PowerWater Kit (Qiagen), and Methods 6 and 7 used phenol-chloroform extraction. An aliquot of a *Chloromonas typhlos* culture was included as a positive control (+c), and extraction blanks were included as negative controls (-c).

Quality assessment of low-biomass samples via agarose gel electrophoresis revealed faint high-molecular-weight bands in the commercial methods, while more distinct bands were observed in Methods 6 and 7 (Fig. 2). Smearing following ultrasonication in Methods 3, 5, and 7 suggests that DNA degradation is associated with this procedure.

Quality assessment of high-biomass samples via agarose gel electrophoresis revealed high-molecular-weight bands in extraction Methods 1, 4, and 6 (Fig. 2). In Method 2, the use of homogenization enhanced the intensity of the high-molecular-weight band, although some DNA degradation was evident. More prominently than in low-biomass samples, ultrasonication led to the appearance of smearing extending into the low-molecular-weight region, indicating substantial DNA degradation due to aggressive lysis. These results were also observed in positive controls. Phenol-chloroform extractions also showed lower-molecular-weight bands (~600 bp), which were also observed in the positive control, indicating that this feature was method-specific rather than sample-dependent.

### Amplicon sequencing: alpha diversity

A total of 8,192,474 paired-end reads were obtained from the 18S rRNA gene amplicons, with an average of 546,165 reads per sample (Table S2). After quality control filtering, 5,988,987 reads remained, averaging 399,266 per sample. Among all methods, Method 7 yielded the highest number of sequences (566,380 ± 29,257), with significant differences compared to the DNeasy PowerSoil Pro commercial kit using vortexing (Method 1; 322,781 ± 84,807) and ultrasonication (Method 3, 312,691 ± 103,769). In terms of total OTU richness, no significant differences were detected among methods, with average OTU counts of 228, 254, 197, 212, and 199 for Methods 1, 2, 3, 6, and 7, respectively (Fig. S1a, Table S3). Shannon and Simpson indices showed significant differences between Method 1 (1.90 and 0.70, respectively) and Method 6 (2.33 and 0.81, respectively), as well as between Methods 6 and 7 for the Shannon index (1.91).

For the 16S rRNA gene amplicons, sequencing yielded 7,149,709 paired-end reads, with an average of 476,647 reads per sample (Table S2). After denoising, merging, and removing chloroplast sequences, 5,463,952 high-quality reads remained, averaging 364,263 reads per sample. Significant differences were observed between the use of ultrasonication in the commercial kit (Method 3) and the phenol-chloroform (Method 7), with mean read counts of 295,487 ± 31,472 and 461,102 ± 3,461, respectively. OTU richness varied across methods, with averages of 208, 216, and 251 for the commercial kit using vortexing (Method 1), homogenization (Method 2), and ultrasonication (Method 3), respectively. The non-commercial methods yielded 151 (Method 6) and 161 (Method 7) OTUs (Fig. S1b). Significant differences were observed between Method 3 and Method 6 (Table S3). Regarding diversity indices, the commercial methods using vortexing and homogenization (Methods 1 and 2) resulted in significantly higher Shannon and Simpson index values, compared to the other approaches. Shannon index values averaged 2.73, 2.75, 2.60, 2.53, and 2.56, respectively, while Simpson index values were 0.89, 0.90, 0.87, 0.87, and 0.87.

### Amplicon sequencing: community composition and beta diversity

**Fig 3:**
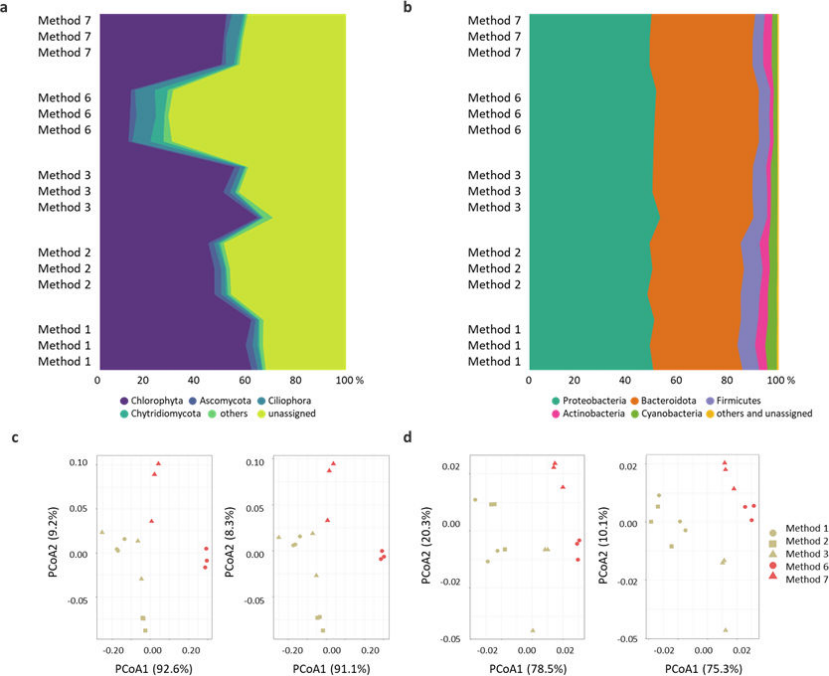
18S and 16S community composition and structure. Eukaryal (**a**) and bacterial (**b**) community composition (relative abundance, %) for each DNA extraction method based on 18S and 16S rRNA gene sequencing. PCoA ordinations of the 18S (**c**) and 16S (**d**) data sets are summarized at the phylum and family levels.

The relative abundance of the main eukaryotic phyla varied depending on the extraction methods used (Fig. 3a). On average, Chlorophyta was the predominant group, with abundance ranging from 61.5% in Method 1 to 46.7% in Method 2. However, in Method 6, green algae represented only 13.8% of the total community. Conversely, Chytridiomycota reached 4.6% in Method 6, 1% in Method 7, and did not exceed 0.7% in Methods 1, 2, and 3. A similar pattern was observed for Ciliophora, which had a relative abundance of 7.6% in Method 6, 4.2% in Method 7, and less than 2% in Methods 1, 2, and 3. Unassigned sequences ranged from 32.8% to 47.1% of the total in Methods 1 and 2, respectively, reaching 69.8% in Method 6.

Beta diversity analyses were performed by grouping samples according to different factor combinations and evaluating community composition at three taxonomic levels: phylum, family, and OTU. Samples were first grouped by DNA extraction method (*n* = 5). At the phylum level, PERMANOVA detected significant heterogeneity in eukaryotic community composition among methods (F = 8.912; *P <* 0.05). Consistent with this result, PCoA revealed clear method-driven separation primarily along the first axis, which explained 92.6% of the total variation, with Method 2 exhibiting the strongest divergence relative to Methods 1 and 3, which clustered more closely together, and phenol–chloroform–based extractions (Methods 6 and 7) also separating from the commercial kit–based approaches, with Method 6 showing a stronger differentiation than Method 7 (Fig. 3c). Hierarchical clustering based on community similarity (Fig. S2a) further supported this separation.

When samples were grouped by extraction technique, comparing commercial (*n* = 3) versus non-commercial approaches (*n* = 2), eukaryotic community composition differed significantly (F = 17.76; *P <* 0.05), consistent with the clearer separation observed in the PCoA ordination. In contrast, grouping samples by the use of ultrasonication for cell lysis (*n* = 2) did not yield significant differences (*P >* 0.05), and samples overlapped in ordination space.

At the family level, PERMANOVA detected significant differences in 18S community composition associated with both extraction method (F = 8.28; *P* < 0.05) and extraction technique (F = 16.02; *P <* 0.05). In agreement with these findings, PCoA ordination showed a pattern highly similar to that observed at the phylum level, with the first axis explaining 91.1% of the total variation and indicating a strong method-driven separation of samples, with the greatest divergence observed for Method 6 relative to all other approaches (Fig. 3c). In contrast, no significant differences were detected at the OTU level, consistent with high variability among replicates and a lack of clear separation in ordination space.

The bacterial community composition was broadly similar across samples (Fig. 3b), with Proteobacteria dominating all extraction methods (48.6% in Method 2 to 50.7% in Method 3), followed by Bacteroidota (35.1% in Method 1 to 41.4% in Method 6) and Firmicutes (4.1% in Method 7 to 7.5% in Method 2). Unclassified sequences and phyla contributing less than 1% of the total community accounted for less than 0.6% of bacterial reads in all samples.

At the phylum level, PERMANOVA indicated significant differences in bacterial community composition among DNA extraction methods (*n* = 5; F = 5.62; *P <* 0.05). Consistent with this result, PCoA showed separation among methods primarily along the first axis, which explained 78.5% of the total variation, with Method 3 (commercial kit with sonication) diverging from Methods 1 and 2 (commercial kits without sonication), which clustered closely together, and Method 7 (phenol-chloroform with sonication) showing the greatest divergence from all other approaches; one replicate of Method 3 exhibited greater within-method variability in ordination space (Fig. 3d). Bray-Curtis hierarchical clustering supported this pattern, identifying Method 7 as a distinct branch, while the remaining methods exhibited high similarity (Fig. S2b). When samples were grouped by extraction technique (commercial vs. non-commercial; *n* = 2), significant differences in bacterial community composition were observed (F = 8.60; *P* < 0.05), whereas grouping based on the use of sonication did not yield significant differences.

A comparable pattern emerged at the family level, where PERMANOVA revealed significant effects of extraction method (F = 6.29; *P* < 0.05) and extraction technique (F = 9.39; *P* < 0.05) on bacterial community composition, while no significant differences were detected between sonicated and non-sonicated samples. In line with these results, PCoA showed separation among samples primarily along the first axis, which explained 75.3% of the total variation. Methods 1 and 2 clustered closely together, indicating similar community profiles, whereas Methods 6 and 7 were distinctly separated from this cluster. Method 3 showed an intermediate pattern (Fig. 3d). At the OTU level, PERMANOVA likewise indicated significant differences across extraction methods (F = 6.63; *P <* 0.05). Hierarchical clustering (Fig. S2c) identified Method 7 as forming a distinct branch, while most other samples clustered closely together.

### Extraction methods and the snow algae community

For sequences assigned to Chlorophyta, a total of 2,120,971 paired-end reads were obtained from the 18S rRNA gene amplicons, with an average of 141,398 reads per sample (Table S2). Significant differences were observed between the commercial method using vortexing (Method 1, 167,430 ± 49,948), homogenization (Method 2, 162,703 ± 41,343), and ultrasonication (Method 3, 141,467 ± 32,404) when compared to Method 6 (31,094 ± 5,705). Method 7 yielded the highest number of sequences (204,298 ± 8,202), significantly surpassing Method 6, which had the lowest recovery.

**Fig 4:**
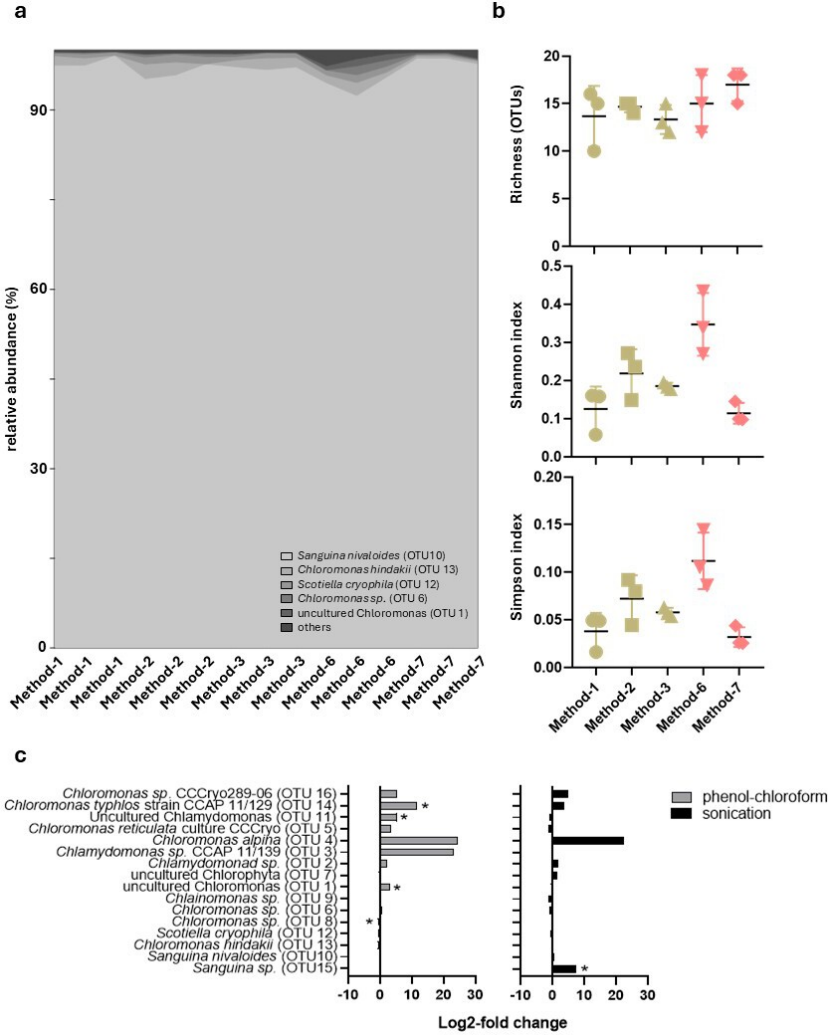
Comparison of DNA extraction methods for sequences assigned to snow algae (Chlorophyta) at the genus level. (**a**) Community composition, (**b**) Alpha diversity, and (**c**) Log2-fold change assessing the effects of phenol-chloroform extraction (Methods 6 and 7 relative to commercial kits, Methods 1–3) and ultrasonication (sonicated Methods 3 and 7 versus non-sonicated Methods 1, 2, and 6) on genus presence and abundance.

Snow algae OTU richness was similar across all methods, with averages ranging from 13 to 17 OTUs (Methods 3 and 7, respectively) (Fig. 4b). Regarding alpha diversity indices, Method 6 showed the highest Shannon and Simpson values (0.35 and 0.11, respectively), with significant differences compared to Method 1 (0.13 and 0.04), Method 3 (0.19 and 0.06), and Method 7 (0.11 and 0.03).

The algal community composition was analyzed at the genus level, revealing a dominance of *Sanguina nivaloides* (OTU 10), which accounted for an average of 96.8% ± 1.8% of the total community (Fig. 4a). This was followed by *Chloromonas hindakii* (OTU 13), representing 1.2% ± 0.8% on average. The remaining genera each contributed, on average, less than 1% of the total community. PERMANOVA analysis at the genus level showed significant differences in community composition among DNA extraction methods (*n* = 5) (F = 21.82; *P <* 0.05). Pairwise comparisons revealed no significant differences between individual methods. Grouping based on extraction technique (*n* = 2) and the use of ultrasonication (*n* = 2) did not yield significant differences.

When assessing the impact of different extraction methods on specific snow algae genera using log₂-fold change (Fig. 4c), log₂-fold changes were calculated relative to the mean abundance obtained with commercial kits (Methods 1–3) to evaluate the effect of phenol-chloroform extraction. Under this comparison, *Chloromonas sp.* (OTU 8) was underrepresented in phenol-chloroform extractions (Methods 6 and 7), whereas *Chloromonas typhlos* CCAP 11/129 (OTU 14), *uncultured Chlamydomonas* (OTU 11), and *uncultured Chloromonas* (OTU 1) were overrepresented. To isolate the effect of ultrasonication, log₂-fold changes were calculated by comparing sonicated versus non-sonicated protocols, revealing significant differences for *Sanguina sp.* (OTU 15), which was detected in Methods 3 and 7 but absent in Methods 1, 2, and 6.

## DISCUSSION

Maximizing DNA recovery from field samples is crucial for obtaining a comprehensive understanding of community composition. However, variations in cell density can influence DNA extraction efficiency, a key consideration given the highly patchy distribution of snow algae blooms (51). To assess this impact, we compared extraction methods using two environmental samples with very different algal abundance. For samples with high snow algae densities, the extraction method used does not appear to be a limiting factor for DNA yield. However, our data suggest that the choice of DNA extraction method significantly influences DNA yield in low-biomass snow samples, which is particularly critical for direct sequencing approaches like metagenomics, as opposed to amplicon sequencing, which includes a PCR amplification step.

Regardless of biomass level, the phenol-chloroform method consistently yielded higher DNA recovery than the commercial kits (Fig. 1). Similar findings have been reported in comparative studies with diverse sample types, including glacier-fed stream sediments (36), biofilms (52), and aquatic microalgae (35). Organic extraction with phenol-chloroform is suggested to minimize DNA loss compared to silica column-based retention used in many commercial kits. However, this method is labor-intensive, is time-consuming, and involves hazardous reagents (35). Although we did not apply additional purification steps, they may be necessary when using non-commercial kits, particularly for high-biomass samples or those rich in airborne organic matter, to remove potential inhibitors. This could also account for the differences observed in the snow algal community, where certain OTUs were under- or over-represented, compared to the commercial methods, which include a built-in purification step. Additionally, the aqueous phase isolation step, where DNA is recovered, is user-dependent, and may introduce variability in DNA yield. In our study, we observed a higher variability in the non-commercial methods, which was more pronounced in low-biomass samples. This procedural variability can affect reproducibility between operators or even across extractions performed by the same individual, whereas commercial kits have been shown to provide more consistent yields across samples (53).

The soil DNA extraction kit outperformed the water kit, indicating that kits designed for more complex matrices may perform better on snow samples, which represent a hybrid matrix of solid water enriched with organic and inorganic material. Although snow generally contains fewer inhibitors than sediments or soils, snowpacks colonized by algae and microbial communities are enriched in extracellular polysaccharides and other biologically derived compounds that can hinder cell lysis and DNA extraction (54), thereby reducing the efficiency of water-specific kits. In addition, snow algae blooms often incorporate mineral dust and carbonaceous aerosols from diverse sources with distinct biogeochemical properties (55, 56), further increasing matrix complexity. By contrast, soil extraction kits are optimized to cope with inhibitory compounds and resilient cells and have proven effective in similarly challenging environments such as permafrost (57).

Regardless of the method used, DNA extraction without ultrasonication resulted in reduced DNA recovery and altered estimates of community richness and composition, an effect that was more pronounced for eukaryotic communities. PERMANOVA and PCoA analyses demonstrate that the lysis strategy is a key driver of eukaryotic community structure, explaining the strong separation of method 6, which lacked an active lysis step (Fig. 3c). This likely reflects incomplete disruption of resistant eukaryotic cells, particularly snow algal cysts. However, at the OTU level, high variability among replicates limited the ability to detect consistent method-driven patterns, resulting in no clear clustering among extraction methods. In contrast, bacterial community composition was influenced more strongly by the overall extraction chemistry (commercial kits versus phenol-chloroform) than by the lysis approach itself (Fig. 3d), consistent with the generally lower resistance of bacterial cells to mechanical disruption. Although cell lysis is a prerequisite for effective DNA recovery, resistance varies widely among microorganisms (58), especially in cold environments where structural adaptations such as thickened or multilayered cell walls are common (59). Snow algal cysts exemplify this strategy, often possessing complex, multilayered walls—including thick secondary layers—that confer resistance to desiccation and freezing stress (60).

At least one snow algae OTU was detected only after applying ultrasonication, further highlighting the resistance of snow algal cysts to lysis and suggesting that standard extraction protocols may underestimate the diversity of snow algae communities. These conclusions apply specifically to community analyses based on 97% OTU clustering, where high variability among replicates limited the detection of consistent method-driven patterns. Higher-resolution approaches, such as zOTUs or ASVs, would likely result in higher diversity estimates (5) and potentially increased variability among replicates rather than clearer differences between methods. However, this was not tested in our study. Therefore, for snow algae samples, particularly low-biomass samples dominated by resistant cyst stages, our data suggest that implementing ultrasonication is highly recommended to enhance DNA recovery and improve the representation of lysis-resistant taxa.

Despite its effectiveness in improving DNA recovery and the detection of lysis-resistant cells, ultrasonication also impacts DNA integrity (Fig. 2). Mechanical shearing and local heating during sonication can fragment genomic DNA into short, non-random fragments, potentially limiting applications that require high-molecular-weight DNA, such as long-amplicon PCR, genome assembly, or metagenomics (61–63). In contrast, short-read amplicon approaches, such as those used in this study, are generally less impacted by DNA fragmentation because they only require a small portion of the target gene to remain intact as a template for PCR amplification (11). However, even short-read amplicon sequencing is not free of methodological risks, as highly fragmented DNA can increase the formation of PCR artifacts such as chimeric products, which, if not carefully controlled for during data analyses, may lead to biased estimates of community diversity (11, 62). Therefore, while ultrasonication is well-suited for amplicon-based community profiling of low-biomass snow algae samples, its use should be carefully evaluated when high molecular weight DNA is required for downstream analyses.

### Conclusion

The choice of DNA extraction kit influenced both DNA recovery efficiency and microbial community characterization. While the extraction method was the primary factor shaping microbial community composition, ultrasonication primarily improved DNA yield in low-biomass samples without significantly altering community structure. Ultrasonication was particularly beneficial for cells that are more resistant to standard lysis methods, including snow algae cysts. At the same time, the benefits of sonication should be balanced against its potential impact on DNA integrity, especially for applications requiring high-molecular-weight DNA. The highest DNA yield and read counts were obtained with a phenol-chloroform extraction combined with sonication, whereas commercial kits yielded greater diversity, particularly for 16S rRNA, based on Shannon and Simpson indices. Considering safety, time, and cost efficiency, we recommend the combination of a soil-specific commercial extraction kit and ultrasonication as a reliable approach, particularly for short-amplicon metabarcoding studies, balancing high DNA recovery with a rapid and effective method for studying snow algae communities.

## Data Availability

Sequence data are archived at the Sequence Read Archive (SRA) at NCBI under BioProject accession no. PRJNA1244840 and BioSample accession no. SAMN47737434 to SAMN47737463.

## SUPPLEMENTAL MATERIAL

Figure S1 aem.00031-26-s0001.tif Diversity indices for the eukaryal community and the bacterial community obtained for the different DNA extraction methods.

Figure S2 aem.00031-26-s0002.tif UPGMA cluster dendrograms based on Bray-Curtis dissimilarity, illustrating the similarities among communities obtained using different DNA extraction methods.

Supplemental legends aem.00031-26-s0003.docx Descriptive legends for Fig. S1 and S2.

Supplemental tables aem.00031-26-s0004.docx Tables S1 to S3.

ASM does not own the copyrights to Supplemental Material that may be linked to, or accessed through, an article. The authors have granted ASM a non-exclusive, world-wide license to publish the Supplemental Material files. Please contact the corresponding author directly for reuse.
