# Supplementary figures and images for: Enhancing DNA recovery in low-biomass snow algae samples: a comparative study of extraction methods and their effect on community composition
Source: Appl Environ Microbiol. 2026 Mar 19;92(4):e00031-26. doi: 10.1128/aem.00031-26 (PMC13101538; doi:10.1128/aem.00031-26)

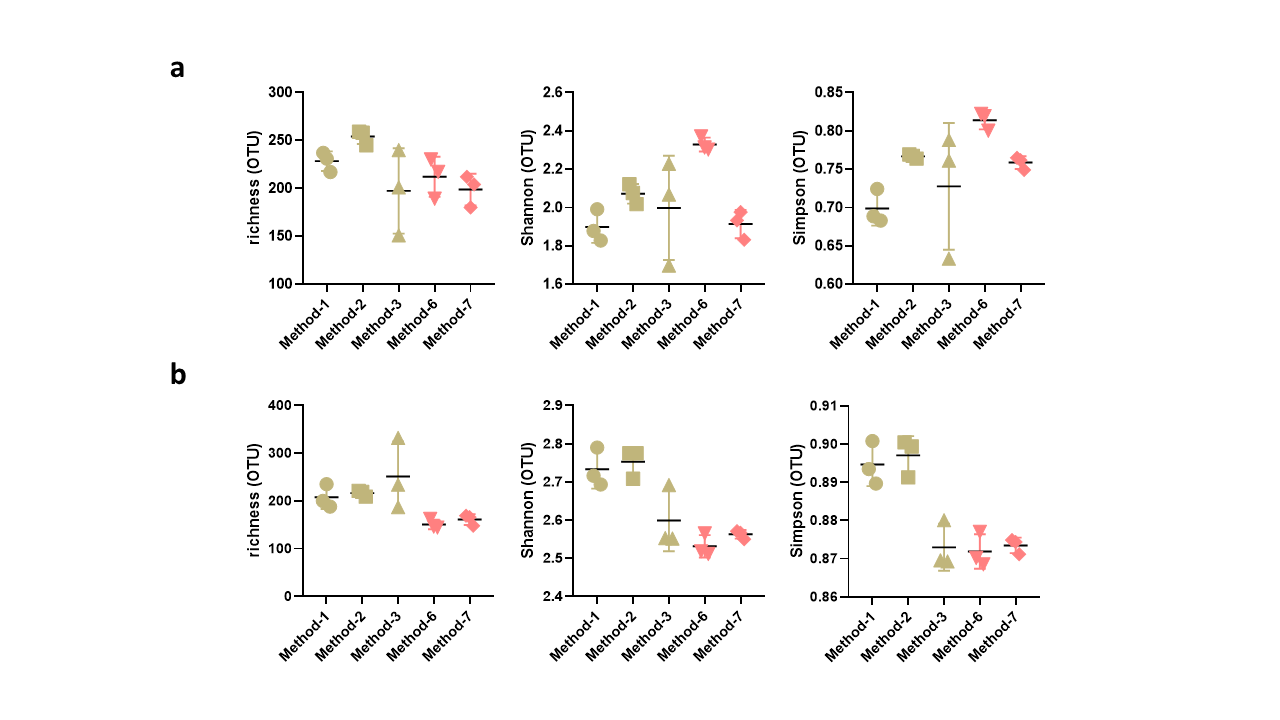

Supplement: Figure S1 — Diversity indices for the eukaryal community and the bacterial community obtained for the different DNA extraction methods. [file aem.00031-26-s0001.tif]

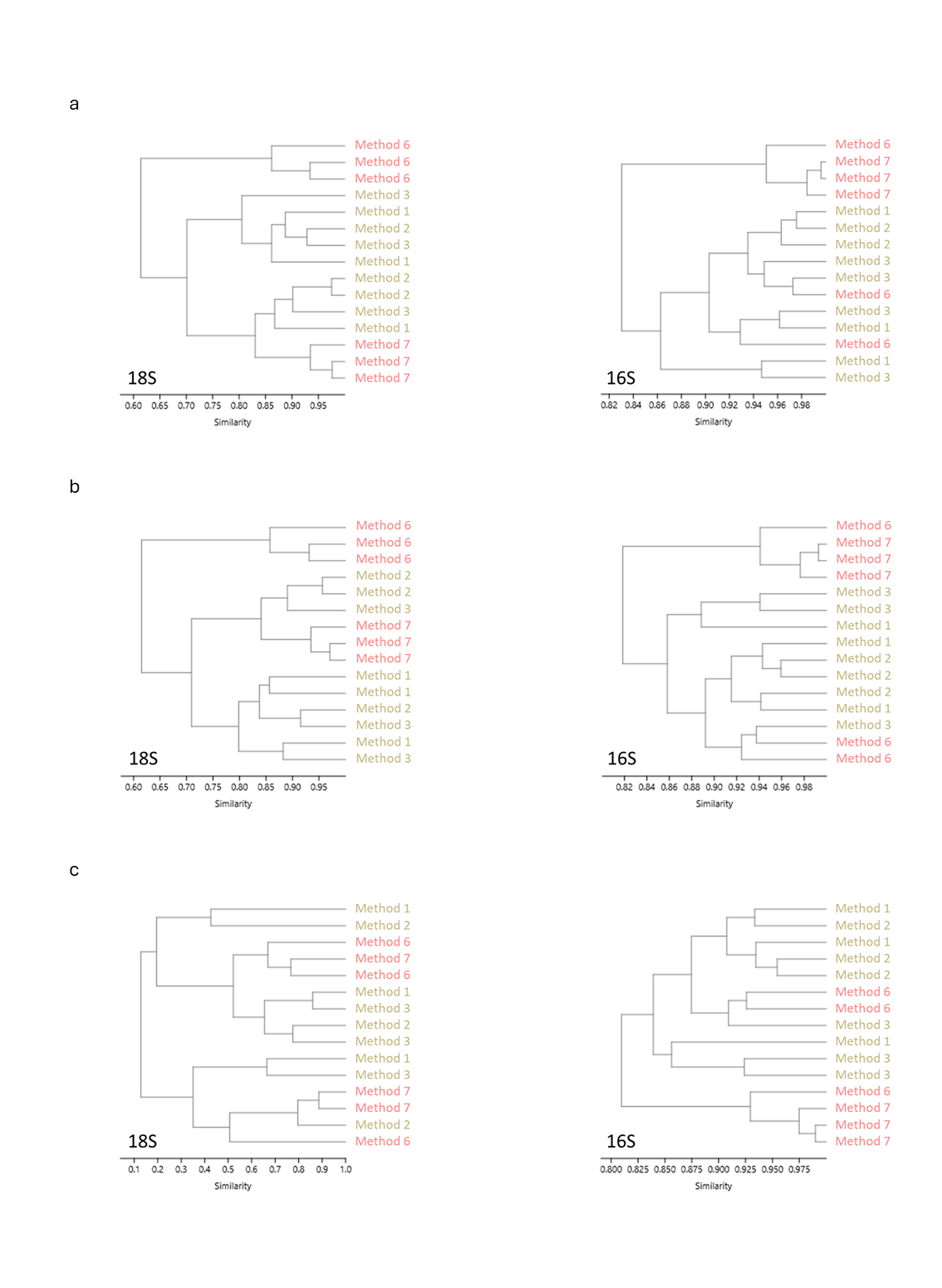

Supplement: Figure S2 — UPGMA cluster dendrograms based on Bray-Curtis dissimilarity, illustrating the similarities among communities obtained using different DNA extraction methods. [file aem.00031-26-s0002.tif]
