# Supplementary material for: Enhancing DNA recovery in low-biomass snow algae samples: a comparative study of extraction methods and their effect on community composition
Source: Appl Environ Microbiol. 2026 Mar 19;92(4):e00031-26. doi: 10.1128/aem.00031-26 (PMC13101538; doi:10.1128/aem.00031-26)
Supplement: Supplemental legends — Descriptive legends for Fig. S1 and S2. [file aem.00031-26-s0003.docx]

# **Supplemental Material**

**Enhancing DNA recovery in low-biomass snow algae samples: a comparative study of extraction methods and their effect on community composition**

PABLO ALMELA and TRINITY L. HAMILTON

*Department of Plant and Microbial Biology, University of Minnesota, St. Paul, Minnesota, USA*

**Supplementary figure 1**. Diversity indices (OTU richness, Shannon index, and Simpson index) for (a) the eukaryal community and (b) the bacterial community obtained for the different DNA extraction methods.

**Supplementary figure 2**. UPGMA cluster dendrograms based on Bray-Curtis dissimilarity, illustrating the similarities among communities obtained using different DNA extraction methods. Dendrograms are shown for the eukaryal and bacterial communities of a snow algae bloom at (a) Phylum level, (b) Family level, and (c) OTU level.
