# Supplementary material for: Enhancing DNA recovery in low-biomass snow algae samples: a comparative study of extraction methods and their effect on community composition
Source: Appl Environ Microbiol. 2026 Mar 19;92(4):e00031-26. doi: 10.1128/aem.00031-26 (PMC13101538; doi:10.1128/aem.00031-26)
Supplement: Supplemental tables — Tables S1 to S3. [file aem.00031-26-s0004.docx]

# **Supplemental Material**

**Enhancing DNA recovery in low-biomass snow algae samples: a comparative study of extraction methods and their effect on community composition**

PABLO ALMELA and TRINITY L. HAMILTON

*Department of Plant and Microbial Biology, University of Minnesota, St. Paul, Minnesota, USA*

**Supplementary Table 1.** Pairwise comparisons of DNA extraction methods based on ANOVA results for samples with (a) low and (b) high snow algae biomass. The table reports whether differences between methods were statistically significant, with significance levels indicated as follows: ns, not significant; * *P < 0.05*; *** *P < 0.001*; **** *P < 0.0001*.

a)

| ANOVA | Significant? | Summary |
| --- | --- | --- |
| Method 1 vs. Method 2 | No | ns |
| Method 1 vs. Method 3 | Yes | *** |
| Method 1 vs. Method 4 | No | ns |
| Method 1 vs. Method 5 | Yes | *** |
| Method 1 vs. Method 6 | Yes | ** |
| Method 1 vs. Method 7 | Yes | **** |
| Method 2 vs. Method 3 | Yes | *** |
| Method 2 vs. Method 4 | No | ns |
| Method 2 vs. Method 5 | Yes | *** |
| Method 2 vs. Method 6 | Yes | ** |
| Method 2 vs. Method 7 | Yes | **** |
| Method 3 vs. Method 4 | Yes | *** |
| Method 3 vs. Method 5 | No | ns |
| Method 3 vs. Method 6 | No | ns |
| Method 3 vs. Method 7 | Yes | * |
| Method 4 vs. Method 5 | Yes | *** |
| Method 4 vs. Method 6 | Yes | *** |
| Method 4 vs. Method 7 | Yes | **** |
| Method 5 vs. Method 6 | No | ns |
| Method 5 vs. Method 7 | Yes | ** |
| Method 6 vs. Method 7 | Yes | ** |

b)

| ANOVA | Significant? | Summary |
| --- | --- | --- |
| Method 1 vs. Method 2 | Yes | **** |
| Method 1 vs. Method 3 | Yes | **** |
| Method 1 vs. Method 4 | No | ns |
| Method 1 vs. Method 5 | Yes | *** |
| Method 1 vs. Method 6 | Yes | **** |
| Method 1 vs. Method 7 | Yes | **** |
| Method 2 vs. Method 3 | Yes | **** |
| Method 2 vs. Method 4 | Yes | **** |
| Method 2 vs. Method 5 | Yes | ** |
| Method 2 vs. Method 6 | Yes | ** |
| Method 2 vs. Method 7 | Yes | **** |
| Method 3 vs. Method 4 | Yes | **** |
| Method 3 vs. Method 5 | Yes | **** |
| Method 3 vs. Method 6 | No | ns |
| Method 3 vs. Method 7 | Yes | * |
| Method 4 vs. Method 5 | Yes | **** |
| Method 4 vs. Method 6 | Yes | **** |
| Method 4 vs. Method 7 | Yes | **** |
| Method 5 vs. Method 6 | Yes | **** |
| Method 5 vs. Method 7 | Yes | **** |
| Method 6 vs. Method 7 | Yes | *** |

**Supplementary Table 2.** Sequencing results for low-biomass samples, showing sequence counts before and after quality filtering and the proportion of high-quality reads obtained with each DNA extraction method for eukaryotic, algal (Chlorophyta), and bacterial communities in a snow algae bloom.

|  | 18S | | | | | Chlorophyta | | | 16S | | | | |
| --- | --- | --- | --- | --- | --- | --- | --- | --- | --- | --- | --- | --- | --- |
|  | input | output | % of input | avg | SD | output | avg | SD | input | output | % of input | avg | SD |
| Method 1 | 555,364 | 412,626 | 74.3 | 322,781 | 84,807 | 218,732 | 167,430 | 49,948 | 544,770 | 414,630 | 76.1 | 345,895 | 70,617 |
|  | 337,268 | 244,121 | 72.4 |  |  | 118,956 |  |  | 370,620 | 273,535 | 73.8 |  |  |
|  | 432,688 | 311,597 | 72.0 |  |  | 164,601 |  |  | 462,203 | 349,519 | 75.6 |  |  |
| Method 2 | 639,803 | 473,353 | 74.0 | 418,275 | 100,507 | 183,198 | 162,703 | 41,343 | 437,502 | 330,173 | 75.5 | 356,225 | 28,179 |
|  | 655,425 | 479,203 | 73.1 |  |  | 189,794 |  |  | 498,689 | 386,133 | 77.4 |  |  |
|  | 410,924 | 302,269 | 73.6 |  |  | 115,116 |  |  | 462,409 | 352,368 | 76.2 |  |  |
| Method 3 | 283,814 | 218,303 | 76.9 | 312,691 | 103,769 | 127,859 | 141,467 | 32,404 | 386,222 | 294,427 | 76.2 | 295,487 | 31,472 |
|  | 413,204 | 295,961 | 71.6 |  |  | 118,085 |  |  | 423,334 | 327,475 | 77.4 |  |  |
|  | 573,323 | 423,808 | 73.9 |  |  | 178,456 |  |  | 342,211 | 264,558 | 77.3 |  |  |
| Method 6 | 442,585 | 311,976 | 70.5 | 376,202 | 60,931 | 24,768 | 31,094 | 5,705 | 469,460 | 358,474 | 76.4 | 362,609 | 53,897 |
|  | 576,804 | 383,438 | 66.5 |  |  | 32,663 |  |  | 407,634 | 310,899 | 76.3 |  |  |
|  | 594,725 | 433,191 | 72.8 |  |  | 35,850 |  |  | 541,245 | 418,455 | 77.3 |  |  |
| Method 7 | 751,414 | 532,674 | 70.9 | 566,380 | 29,257 | 198,481 | 204,298 | 8,202 | 603,795 | 460,587 | 76.3 | 461,102 | 3,461 |
|  | 763,440 | 581,267 | 76.1 |  |  | 200,733 |  |  | 604,215 | 457,927 | 75.8 |  |  |
|  | 761,693 | 585,200 | 76.8 |  |  | 213,679 |  |  | 595,400 | 464,792 | 78.1 |  |  |

**Supplementary Table 3.** Results of Tukey's multiple comparisons test for diversity indices across different DNA extraction methods for (a) eukaryotic and (b) prokaryotic communities of a snow algae bloom. Comparisons were performed for OTU richness, Shannon index, and Simpson index. Significant levels are indicated as follows: ns, not significant; * *P < 0.05*; *** *P < 0.001*; **** *P < 0.0001*.

a)

|  | Richness (OTUs) | | Shannon Index | | Simpson Index | |
| --- | --- | --- | --- | --- | --- | --- |
| Tukey's multiple comparisons test | Significant? | Summary | Significant? | Summary | Significant? | Summary |
| Method 1 vs. Method 2 | No | ns | No | ns | No | ns |
| Method 1 vs. Method 3 | No | ns | No | ns | No | ns |
| Method 1 vs. Method 6 | No | ns | Yes | * | Yes | * |
| Method 1 vs. Method 7 | No | ns | No | ns | No | ns |
| Method 2 vs. Method 3 | No | ns | No | ns | No | ns |
| Method 2 vs. Method 6 | No | ns | No | ns | No | ns |
| Method 2 vs. Method 7 | No | ns | No | ns | No | ns |
| Method 3 vs. Method 6 | No | ns | No | ns | No | ns |
| Method 3 vs. Method 7 | No | ns | No | ns | No | ns |
| Method 6 vs. Method 7 | No | ns | Yes | * | No | ns |

b)

|  | Richness (OTUs) | | Shannon Index | | Simpson Index | |
| --- | --- | --- | --- | --- | --- | --- |
| Tukey's multiple comparisons test | Significant? | Summary | Significant? | Summary | Significant? | Summary |
| Method 1 vs. Method 2 | No | ns | No | ns | No | ns |
| Method 1 vs. Method 3 | No | ns | Yes | * | Yes | ** |
| Method 1 vs. Method 6 | No | ns | Yes | ** | Yes | ** |
| Method 1 vs. Method 7 | No | ns | Yes | ** | Yes | ** |
| Method 2 vs. Method 3 | No | ns | Yes | * | Yes | *** |
| Method 2 vs. Method 6 | No | ns | Yes | ** | Yes | *** |
| Method 2 vs. Method 7 | No | ns | Yes | ** | Yes | ** |
| Method 3 vs. Method 6 | Yes | * | No | ns | No | ns |
| Method 3 vs. Method 7 | No | ns | No | ns | No | ns |
| Method 6 vs. Method 7 | No | ns | No | ns | No | ns |
